# Prevalence of adolescent idiopathic scoliosis among primary school children in Canakkale, Turkey

**DOI:** 10.1186/1748-7161-7-S1-O37

**Published:** 2012-01-27

**Authors:** H Yilmaz, C Zateri, S Vurur, C Bakar

**Affiliations:** 1 Canakkale Onsekiz Mart University PMR Department, Canakkale, Turkey; 2 Canakkale Onsekiz Mart University Canakkale, Turkey

## Background

Adolescent idiopathic scoliosis (AIS) is an important health problem among school children. There are a few study performed to determine prevalence of AIS. However, reported rates are many different. We aimed to determine prevalence of AIS in Canakkale.

## Materials and methods

The universe of our study was chosen from primary school students. The sample size was calculated separately for provinces and districts, 1321 and 1420, respectively. We chose 12 schools totally by cluster sampling method. Presence of AIS was evaluated with scoliometre and posture analysis. Students who have skeletal deformity or major skeletal surgery history were excluded. SPSS 15.0 was used for analysis.

## Results

We reached a total of 2604 students. We found47 (1.8%) studentswith suspected AIS. All of the suspected students were invited to the hospital to assess with x-ray. 26 (61.7%) of all students came to the hospital and we found 8 students have AIS. The data presented in this study are the preliminary results. Student evaluation is ongoing. We aim to reach all the suspected cases. Then, we will notify the prevalence of AIS among primary school children in Canakkale.

## Conclusions

Idiopathic scoliosis is classified as infantile, juvenile and adolescent depending on the age at first diagnosis. Extremely rare in infancy and early childhood but has a prevalence of 1% to 2% among school children up to age 15. We aimed to determine the frequency of AIS in the risk population of those aged 7-14 years and providing a base for large-scale studies.

